# Juvenile Angiofibroma: Evolution of Management

**DOI:** 10.1155/2012/412545

**Published:** 2011-11-17

**Authors:** Piero Nicolai, Alberto Schreiber, Andrea Bolzoni Villaret

**Affiliations:** Department of Otorhinolaryngology, University of Brescia, Piazza Spedali Civili 1, 25123 Brescia, Italy

## Abstract

Juvenile angiofibroma is a rare benign lesion originating from the pterygopalatine fossa with distinctive epidemiologic features and growth patterns. The typical patient is an adolescent male with a clinical history of recurrent epistaxis and nasal obstruction. Although the use of nonsurgical therapies is described in the literature, surgery is currently considered the ideal treatment for juvenile angiofibroma. Refinement in preoperative embolization has provided significant reduction of complications and intraoperative bleeding with minimal risk of residual disease. During the last decade, an endoscopic technique has been extensively adopted as a valid alternative to external approaches in the management of small-intermediate size juvenile angiofibromas. Herein, we review the evolution in the management of juvenile angiofibroma with particular reference to recent advances in diagnosis and treatment.

## 1. Epidemiology

Juvenile angiofibroma (JA) is a benign vascular neoplasm which affects young males between 9 and 19 years of age and accounts for 0.05% of all head and neck tumors [1]. In USA, this lesion represents the most frequent head and neck tumor of adolescence with one new case per 5000 to 50,000 patients referred to an otolaryngologist [2]. Glad and colleagues [3] reported an incidence of JA in Denmark of 0.4 cases per million inhabitants per year. In the Middle East and India, the incidence seems to be much higher than in Europe [4].

## 2. Histopathological Aspects and Pathogenesis

**Figure 1:**
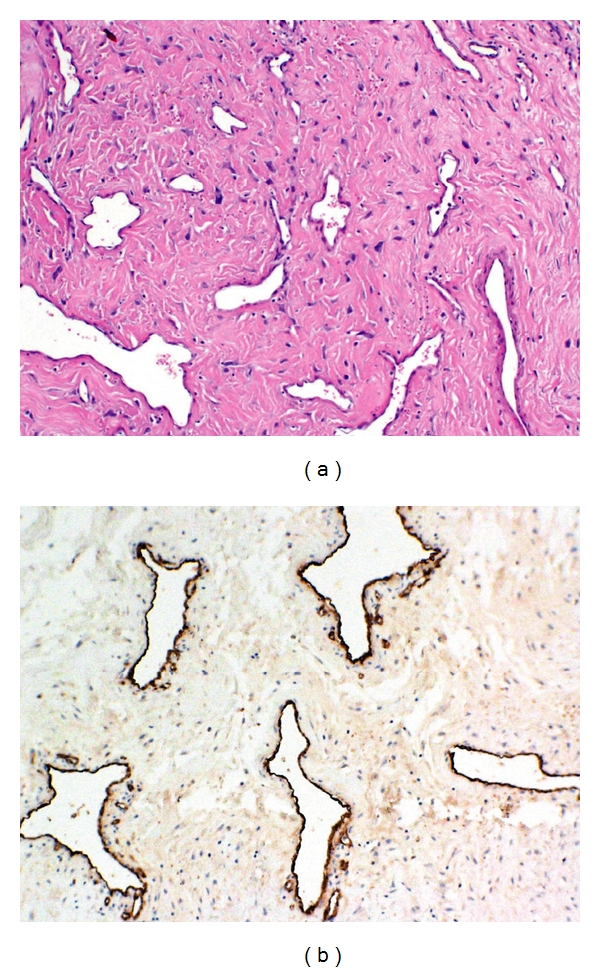
Microscopic appearance of JA (hematoxylin-eosin staining (a) and immunohistochemistry for factor VIII (b)). Vessel caliber is extremely variable, the muscular layer of vessels is frequently absent, and stromal cells have usually a spindle-shaped appearance.

Histologically, JA is a pseudocapsulated lesion characterized by an irregular vascular component composed of numerous blood vessels of different calibers embedded in a fibrous stroma, rich in collagen and fibroblasts. Vessels are slit or dilated, organized in clusters, without elastic fibers in their wall, and the muscular lining is incomplete in large vessels, and totally absent in the smaller ones. Mitotic figures are rare [5, 6] (Figure 1). 

Since the 19th century, there is considerable debate concerning the fibrous or vascular origin of JA. Because of its extensive vascularization, several authors have considered the hypothesis of vasoproliferative malformation: Sternberg [7] and Hubbard [8] proposed JA as a specific type of hemangioma, while the theory of an ectopic proliferating vascular tissue was forwarded by Schiff in 1959 [9]. More recently, immunohistological and electron microscopic studies have suggested that this lesion may be considered a vascular malformation (or hamartoma) rather than a tumor [10]. These observations led Schick and colleagues [11] to postulate that JA might be due to incomplete regression of the first branchial artery, which arises in embryogenesis between days 22 and 24 and forms a temporary connection between the ventral and dorsal aorta. This artery commonly regresses and forms a vascular plexus that either involutes or may leave remnants, potentially leading to development of JA. This theory is supported by the finding that JA vessels express laminin alpha-2, which is considered to be a marker for early embryological angiogenesis [12]. Moreover, Gramann et al. [13] demonstrate prominent collagen-type VI expression in JAs, which is an extracellular matrix component that is attractive for neural crest cells and might be involved in the development of JA from plexus remnants of the first brachial artery.

The observation that JA typically arises in adolescent males and that the lesion frequently regresses only after full development of secondary sex characteristics provided the evidence of hormonal influence on JA growth [14, 15]. In spite of reports of hormonal disorders in patients with JA and the presence of androgen and/or estrogen receptors and their role in the tumor development or regression, a hormonal pathogenesis of this lesion is still a matter of debate [14–19].

Many studies have demonstrated numerous chromosomal alterations in patients affected by JA. Gains at chromosomes 4, 6, 8, and X and losses on chromosomes 17, 22, and Y are the most frequent chromosomal abnormalities detected [11, 20–23]. Moreover, Schick [21] described the gene AURKA (20q13.2), a centrosome-associated serine/threonine kinase, with a possible role in chromosomal and genetic instability in JA. These data provide important information regarding the possible location of tumor suppressor genes and oncogenes potentially involved in the pathogenesis of JA.

The observation of an increased prevalence of JA in patients with familial adenomatous polyposis (FAP) suggested a possible association between these two pathologies [24]. Although evidence of adenomatous polyposis coli (APC) gene mutations was not found [25], activating *β*-catenin gene alterations are frequently detected in JA [26]. The APC proteins regulate the level of *β*-catenin, which play a role in cell-cell adhesion and in the Wnt signaling pathway as a transcriptional activator. Nuclear accumulation of mutated *β*-catenin suggested that APC/*β*-catenin pathway might be involved in JA pathogenesis. Moreover, *β*-catenin can act as coactivator of androgen receptors and consequently increase tumor androgen sensitivity, which might explain why JA develops in adolescent males [21].

Schick et al. [27] and Nagai et al. [28] observed losses of the tumor suppressor gene p53 in 5 of 7 cases and increased expression of p53 mRNA in 32% of patients affected by JA, respectively. Nagai et al. also described increased expression of the oncogene c-fos in 14% of cases. However, further studies are necessary to better understand if the tumor suppressor p53 and oncogenes of the fos family play a role in JA growth.

Renkonen and colleagues [29] investigated the expression of growth factor receptor C-KIT, protooncogene C-MYC, and polycomb protein and oncogene BMI-1 in JA. They observed C-MYC and BMI-1 expression only in stromal cells, whereas C-KIT immunoexpression was shown in both stromal and endothelial cells suggesting that both the stromal and the vascular component may be involved in the neoplastic growth of JA.

The oncogenes Ki-ras, Ha-ras, and Her-2/neu have been investigated with no detection of mutations [27, 30].

## 3. Site of Origin and Patterns of Spread

**Figure 2:**
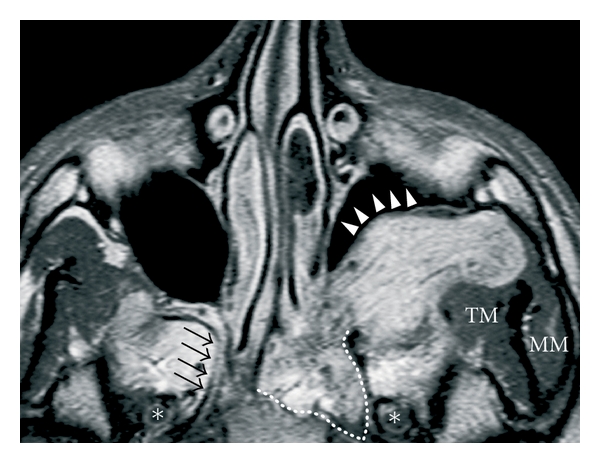
Axial contrast enhanced MRI: extensive JA with a typical pattern of spread into the cancellous bone of the basisphenoid along the vidian canal (white dotted line); on the contralateral side, black arrows indicate the right vidian nerve. Moreover, the lesion spreads deeply into the pterygomaxillary fossa toward the masticatory muscles, with anterior displacement of the posterior maxillary wall (white arrowheads). Asterisks indicate the foramen ovale bilaterally. TM: temporalis muscle; MM: masseter muscle.

**Figure 3:**
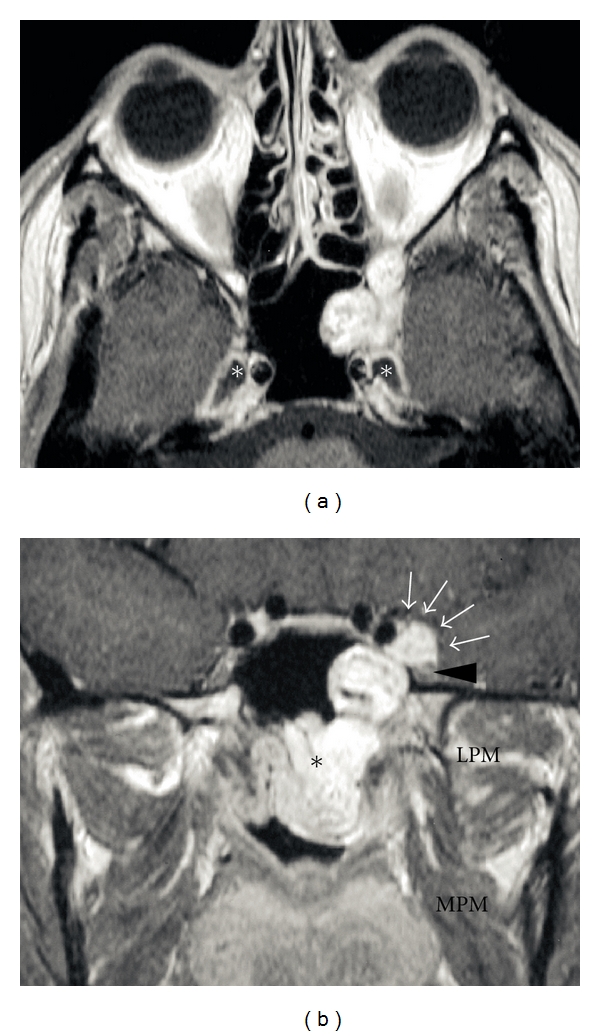
Axial (a) and coronal (b) contrast-enhanced MRI. JA with its epicenter into the root of the left pterygoid process. The nasopharyngeal component with submucosal spread is clearly evident (black asterisk). The lesion reaches the intracranial extradural compartment through the inferior and superior orbital fissures (white arrows), inferolaterally displacing the maxillary nerve (black arrowhead). The white asterisk indicates Meckel's cave. LPM: lateral pterygoid muscle; MPM: medial pterygoid muscle.

JA is considered to arise in the area of the sphenopalatine foramen; based on the results on CT or MR imaging, some authors consider that the lesion originates in the pterygopalatine fossa at the level of vidian canal aperture [31]. The growth of the lesion has the peculiar tendency to follow a submucosal plane, growing in the adjacent anatomical sites that offer less resistance and invade the cancellous bone of the basisphenoid. Because of the constant site of origin and the knowledge of tumor behavior in relation to surrounding tissues, spreading patterns of JA are highly predictable [32, 33]. From the pterygopalatine fossa, the tumor grows medially into the nasopharynx, nasal fossa, and eventually towards the contralateral side. Laterally it can extend into the sphenopalatine and infratemporal fossae, via an enlarged pterygo-maxillary fissure with typical anterior displacement of the posterior maxillary wall, until it comes in contact with masticatory muscles and soft tissues of the cheek (Figure 2). Posterior growth may find several points of minor resistance through which JA may reach critical anatomic structures such as the internal carotid artery (ICA) through the vidian canal, cavernous sinus through the foramen rotundum medially to the maxillary nerve, and the orbital apex through the inferior orbital fissure (Figure 3). Bone involvement occurs via two main mechanisms: (1) resorption by direct pressure of preexisting bony structures with osteoclastic activation or (2) direct spread along perforating arteries into the cancellous root of the pterygoid process. Subsequent extension posteriorly towards the upper-middle of the clivus and laterally within the greater wing of the sphenoid, usually with late erosion of the inner table of the middle cranial fossa, may be detected in advanced cases [34]. Intracranial extension along a canal or resulting from spreading through bone destruction cannot be considered a rare event, especially in large JA, while infiltration of the dura is very rare [35]. 

## 4. Clinical and Radiologic Findings

Typical symptoms for JA are progressive unilateral nasal obstruction (80–90%) with rhinorrhea and recurrent unilateral epistaxis (45–60%), and thus these complaints in a male adolescent should immediately generate suspicion. Headache (25%) and facial pain may arise secondarily to the blockage of paranasal sinuses, or impairment of Eustachian tube function with unilateral secretory otitis media, respectively. Tumor extension into the sinonasal cavity can cause chronic rhinosinusitis. Proptosis and alteration of the vision clearly indicate an involvement of the orbit. Swelling of the cheek, neurologic deficits, alteration in olfaction, rhinolalia clausa, and otalgia are also possible [1].

**Figure 4:**
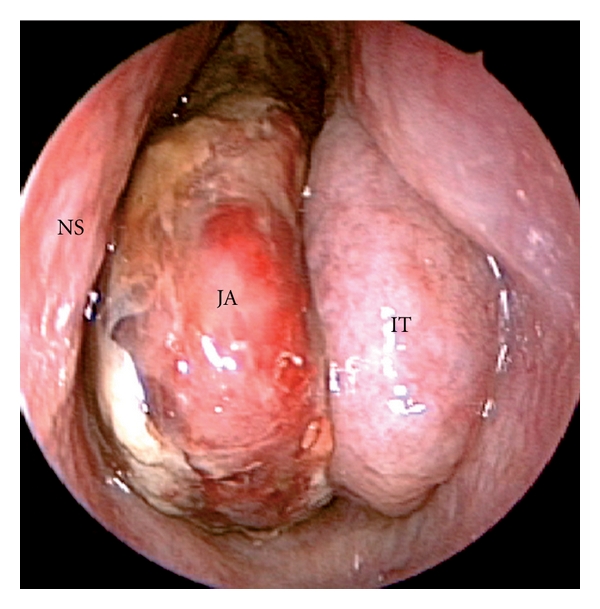
Endoscopic appearance of JA showing a lobulated hypervascularized mass with a smooth surface partially covered by fibrin growing into the left nasal fossa. NS: nasal septum; IT: inferior turbinate; JA: juvenile angiofibroma.

Given the presenting symptoms, the patient should be examined by nasal endoscopy which usually shows a large, lobulated mass behind the middle turbinate filling the choana with a smooth surface and clear signs of hypervascularization (Figure 4). 

Since epidemiologic and endoscopic findings are typical, biopsy is absolutely contraindicated because of a considerable and undue risk of massive hemorrhage [1].

Imaging techniques after contrast enhancement (MSCT and/or MR) are crucial to confirm the clinical suspicion pattern of vascularization and to assess the extension of the lesion. The diagnosis on imaging is based on three factors: the site of origin, hypervascularization after contrast enhancement, and patterns of growth [31, 32]. The evidence that up to 96% of JA caused enlargement or erosion of the anterior part of the vidian canal supports the hypothesis of its typical location in the pterygopalatine fossa at the exit of this canal [32]. After administration of contrast agent, a strong and homogeneous enhancement on MSCT or MR is visible, with several signal voids within the lesion in both MR T1 and T2 sequences, indicating major intralesional vessels [31]. Moreover, enlargement of the internal maxillary artery can be detected by MSCT or MR, as well as signs of bone remodeling whereby thinning and anterior displacement of the posterior maxillary wall, with bone erosion typically at the level of the pterygoid root. Without doubt, MR better depicts cancellous bone invasion and, in lesions invading the middle cranial fossa, the relationship of the lesion with cavernous sinus and dura. Differential diagnosis includes other hypervascularized lesions such as hemangiopericytoma, lobular capillary hemangioma, and paraganglioma which have a different gender/age distribution and pattern of growth.

Preoperative identification of blood supply is a crucial finding to select the most appropriate surgical strategy. Although angio-MR may help in the vascular assessment, the complete map of all feeders requires digital subtraction angiography. Typically the JA receives vascular supply via the external carotid system and particularly from internal maxillary, ascending pharyngeal, and vidian arteries [1]. Vascular components from branches of ICA, such as the inferomedial trunk or inferior hypophyseal artery, may be frequently detected in large lesions involving the skull base and in contact with ICA. Because of the frequent detection of bilateral vascular supply, around 36% by Wu et al. [36] in a recent literature review, both carotid systems require angiographic evaluation.

Preoperative embolization is recommended by most authors [37–40] as a standard procedure to reduce blood loss during surgical resection. Some reports [33, 41] have stated that this procedure did not affect perioperative bleeding, although some years later Glad et al. [3] observed that embolization provides a 60–70% reduction in intraoperative bleeding, and the need for blood transfusion is required. Although the modification within the lesion induced by the embolization has been indicated as a contributory cause of incomplete excision [42], refinements in the technique and the introduction of new materials have minimized the risk of leaving residual disease. During the last decade, the availability of small particles and microcatheters has made it possible to reach collateral and terminal branches of external carotid artery to avoid the risk of neurologic sequelae following the inadvertent embolization of small vessels supplied by the ICA. Polyvinyl-alcohol particles are the most frequently used material for this procedure, which must be planned 24–48 hours before surgery to avoid the risk of revascularization [1]. As suggested by Hackman et al. [43], when vessels from both external carotid systems vascularize the lesion, bilateral embolization of internal maxillary artery is recommended. To control bleeding arising from vessels supplied by the ICA in lesions with extensive skull base involvement, Tranbahuy et al. [44] introduced a technique of direct embolization through a transnasal or lateral transcutaneous access with a mixture of cyanoacrylate, Lipiodol, and tungsten powder. In view of the possible occurrence of severe neurologic complications, this technique has not gained much popularity [45]. However, some recent reports on limited numbers of patients treated with a new embolic material, Onyx, with properties that seem to prevent its migration, have revived interest in this procedure [46, 47]. In the rare instance of huge lesions with ICA encasement, balloon occlusion test and sacrifice of this vessel may be considered [45].

## 5. Staging Systems

Different staging systems based on tumor extension have been proposed to stratify patients with the intent of easing comparison between different series. Over the years, several authors have modified and adapted staging systems based on advances in diagnostic and treatment techniques. Since 1981, when the first staging system was introduced by Sessions et al. [48], many other systems have been used. [2, 49–51] Only those proposed by Andrews et al. [52] and Radkowski et al. [53] have been quite extensively adopted. More recently, the staging systems proposed by Önerci et al. [54], Carrillo et al. [55], and Snyderman et al. [56] attempted to give indications concerning treatment planning by identifying lesions amenable to endoscopic surgery and those resectable by an external or combined approach. Additionally, Snyderman et al. [56] introduced a new parameter useful in preoperative evaluation represented by residual vascularity after embolization.  Table 1 summarizes the most common classifications used in clinical practice.

## 6. Surgical Treatment

Although several nonsurgical methods have been proposed, surgery is unanimously considered the treatment of choice for JA. In the last two decades, the surgical approach to the lesion has considerably evolved mainly in relation to the indication of endoscopic techniques. Transpalatal, transpharyngeal, transfacial through lateral rhinotomy, midfacial degloving, and Le Fort I osteotomy, other than infratemporal and subtemporal lateral approaches [39, 57, 58] were once the traditional surgical methods commonly performed to remove JA. Advances in radiological imaging and improvements of embolization techniques have significantly contributed to better preoperative management and treatment planning. Moreover, increasing experience in endoscopic surgery together with better understanding of complex sinonasal anatomy, the possibility to safely reach adjacent sites through the nose such as the orbit, infratemporal fossa, masticatory space, parasellar region, the availability of navigation systems, and the well-known morbidity associated with external procedures have made an endoscopic approach a viable alternative. Due to the fact that one of the most challenging aspects in JA surgery is control of intraoperative bleeding, the cooperation of an anesthesiologist with endoscopic skull base experience, the availability of a cell salvage machine and any material (absorbable gelatin powder, sponge oxidized regenerated cellulose, microfibrillar collagen, fibrin, or synthetic sealants) [59] that helps the surgeon to control bleeding are mandatory.

In the 1990s, several authors reported their first experience of transnasal endoscopic resections for early stage JA, demonstrating the feasibility of this procedure and recurrence rates similar to that observed with external approaches, in addition to lower risk and morbidity [37, 57, 60–66]. Nicolai et al. [67] in 2003 reported that lesions extending to the nasopharynx, nasal cavities, sphenoid sinus, ethmoid sinus, maxillary sinus, and/or pterygopalatine fossa could be managed successfully through endoscopic surgery [67]. There is no doubt about the role of the surgeon's experience and “learning curve” in JA management with consequent widening of indications for an endoscopic approach, from early stage to lesions staged IIC and IIIA, according to the classifications of Radkowsky and Andrews, respectively, [67–75]. More recently, Mohammadi Ardehali et al. [76] asserted that endoscopic resection of JA is strongly recommended as a first surgical step for tumors with stages (I) to (III-A) of Radkowsky's staging system because of its significant lower intraoperative blood loss, hospitalization, and recurrence rate in comparison to traditional approaches. Furthermore, several series suggested that this technique can be performed even in JAs that extend to the infratemporal fossa, orbit, and/or parasellar region, compatible with the capabilities and experience of the surgeon [39, 44, 76, 77]. A crucial issue is represented by those lesions with large infiltration of skull base, extensive vascular supply from ICA, or encasement of the artery itself: an anterior or lateral combined external approach according to the relationship of the tumor with ICA, and the surgeon's preference should be planned. Moreover, endoscopic surgery is contraindicated in residual tumors involving critical areas (ICA, optic nerve, cavernous sinus, dura), whereas adhesions due to scar tissue increase the risk of severe uncontrolled complications during dissection of the lesion [77].

The first surgical step when the surgeon approaches a JA endoscopically is to expose the tumor as extensively as possible through a middle turbinectomy, ethmoidectomy, wide antrostomy and sphenoidotomy, and resection of the posterior third of the nasal septum, which enhances the exposure of the nasopharyngeal portion of the lesion. The posterior wall of the maxillary sinus has to be resected as far lateral as dictated by the lateral extension of the lesion into the pterygopalatine and/or infratemporal fossae. For JA largely involving the infratemporal fossa, the surgeon can improve lateral exposure through a so-called Sturmann-Canfield maxillectomy, which provides resection of the anteromedial corner of the maxillary sinus [77]. An endoscopically assisted antral window approach through the anterior wall of the maxillary sinus, as proposed by Pasquini et al. [72], may be considered a possible alternative. Another important principle in the resection of large-volume lesions is the fragmentation technique (“piece-meal” resection) that helps to completely assess the extension without an increased risk of recurrence [69]. During dissection, to maintain a proper cleavage plain between the tumor and adjacent tissues, a four-handed technique is highly recommended [77]. The procedure is completed by accurate subperiosteal dissection of the tumor attachment and subsequent extensive drilling of the basisphenoid and other bone area where the JA is adhered to remove residual disease, which may not be immediately evident, and prevent its regrowth [78].

Because of its high degree of vascularization, bleeding during surgery is a crucial topic. Some studies compared the blood loss between endoscopic and external approaches, showing a lower loss in endoscopic surgery [78, 79]. However, the reliability of these data requires confirmation since JAs treated by an open approach usually have a higher stage than those resected endoscopically. Another question widely discussed in literature is the reduction of intraoperative bleeding, thanks to preoperative embolization. Some authors have correlated the amount of blood loss with the quality of embolization and with tumor extension [67, 71]. Glad et al. [3] showed a statistically significant decrease in bleeding between the nonembolized (650 mL) and the embolized group (1200 mL).

To better control bleeding during the procedure, several authors have proposed the use of diode laser, KTP laser, or ultrasonic scalpel [70, 77, 80–82].

## 7. Postoperative Surveillance

Based on the experience by Kania et al. [83], Lund et al. [1], and Nicolai et al. [77] recommended postoperative MR imaging after removal of the nasal packing and until 72 hours for early identification of any suspicious residual disease. The reason for this is the presence of minor inflammatory changes, typically observed 3-4 months after treatment, which frequently challenge differentiation between residual JA and active scar tissue. Although this surveillance policy has to be validated by longer follow-up periods, Nicolai et al. [77] observed that patients with no signs of persistence do not develop any lesion even at subsequent MR examination. Endoscopic examination has limited value in the identification of residual/recurrence disease because of the submucosal growing pattern of JA, which is detected with more precision by enhanced MR or MSCT. Whatever technique is selected, the examination should be performed every 6–8 months for at least 3 years after surgery. Moreover, depending on suspicious enhancement, incomplete resection and age of onset, angio-MR imaging may be scheduled [1]. Closer radiologic survey may be required to better evaluate the growth and plan treatment for persistent JA.

## 8. Outcome

Although comparison between external approaches and endoscopic techniques is biased by the different staging systems and follow-up strategy adopted, recently Wang et al. [84] observed no significant difference in the rate of recurrence between 11 patients treated endoscopically and 13 who underwent transpalatal excision, all staged (I) and (II) according to Chandler classification. Series with a consistent number of patients treated with external approaches have shown a reduction over time in terms of recurrence rate ranging from 36–40% [34, 85] reported in the 1990s, to the excellent results of Danesi et al. [35] in 2008 with 13.5% and 18.2% of residual disease in lesions with extracranial and intracranial extension, respectively. At present, endoscopic resection in small/intermediate JA is widely recommended because of the low risk of recurrence demonstrated in several studies during the last decade [34, 71, 73, 85–89].

Currently, the results of the two major series (Table 2) of JA resected through an endoscopic approach corroborate the principle that this modality of treatment can encompass all lesions from stage (I) to (IIIA) or (IIIB) according to Radkowsky and Andrews staging systems, respectively [76, 77]. An overall recurrence/residual rate of 8.6% [77] and 19.1% [76], respectively, was reported. As previously highlighted by Howard et al. [78], Nicolai et al. [77] in their study on 46 patients treated with an exclusive endoscopic procedure observed that all the residual lesions detected in 4 patients by MR within 24 months after treatment were located at the level of the basisphenoid bone, thus emphasizing the need to extend drilling well beyond the apparent margin of tumor infiltration.

In a recent study on 95 JA, Sun et al. [90] identified three predictive factors that may increase the recurrence rate: patient age at diagnosis (under 18 years), tumor size (>4 cm), and stage according to Radkovsky classification.

The management of residual disease, especially when located intracranially or with a relationship with critical structures such the ICA, cavernous sinus, and the optic nerve, remains a source of discussion. Certainly, close survey with enhanced MR or MSCT is strongly indicated to evaluate the growth rate, and consequently, the need for surgical revision. Önerci et al. [54] prefer an observational strategy to infratemporal craniofacial resection for intracranial residual disease. Moreover, some reports [91–93] described the possibility, in at least a minority of cases, of spontaneous involution or reduction in size because of hormonally dependent JA pathogenesis.

## 9. Nonsurgical Treatments

The use of radiation therapy (RT) in JA is still debated for the reported risk of sarcomatoid transformation [94] or radio-induced neoplasms in the following decades. Some authors recommended RT as adjuvant treatment in unresectable tumors, in failure of complete tumor removal, or for extensive intracranial extension [53, 95, 96]. Nicolai et al. [77] suggested that RT may be indicated for residual lesions in critical areas that have been demonstrated to increase in size. Lee et al. [97] reported on 27 patients affected by advanced JA treated primarily with RT (30–55 Gy): the recurrence rate was 15%, and long-term complications observed in 4 patients included growth retardation, panhypopituitarism, temporal lobe necrosis, cataracts, and radiation keratopathy. More recently, McAfee et al. [98] treated 22 patients affected by high staged JA with RT (30–36 Gy): in 10 cases as primary treatment, and in 12 for recurrence. Local control was obtained in 90% of patients, with 2 cases of local persistence. Late complications, which occurred in 7 (32%) cases, included cataracts, transient central nervous system syndrome, and cutaneous basal cell carcinoma. The use of intensity-modulated RT for the treatment of 3 patients with extensive or persistent JA showed no recurrences and a late toxicity with epistaxis and chronic rhinitis in 2 cases [99]. Although a small number of cases of JA have been treated by Gamma-Knife radiosurgery [100, 101], the insignificant morbidity documented may be reasonably correlated to the optimized irradiation of the target volume by sparing uninvolved structures.

The use of chemotherapy in treatment of JA is supported by only a few reports [102–104]. In the 1980s, Goepfert et al. [102] described successful results with two different chemotherapy schedules, including doxorubicin and dacarbazine, or vincristine, dactinomycin, and cyclophosphamide.

Several studies on hormone pathogenesis have extensively demonstrated the hormonal dependence of this tumor, suggesting a promising role of estrogen or androgen receptor blockers in its treatment [9, 15–19]. Gates et al. administered flutamide, a potent nonsteroidal androgen receptor blocker, in 5 patients affected by JA and detected an average tumor regression of 44% in four cases [105]. However, in a report on 7 patients, Labra et al. observed no significant differences between tumor dimensions before or after flutamide administration, questioning its use in JA [106]. Very recently, flutamide-induced regression in a series of 20 advanced staged JA was demonstrated only in postpuberal patients [107].

## 10. Conclusions

Juvenile angiofibroma is a pathology that should be included in the differential diagnosis of unilateral nasal obstruction, associated or not with epistaxis, especially in young adolescent males. The finding at nasal endoscopy, which is the first step in the diagnostic algorithm, of a hypervascularized lesion occupying the posterior half of the nasal fossa should immediately raise suspicion. Morphologic imaging confirms the diagnosis. Endoscopic surgery after embolization has been demonstrated to be a viable alternative to external techniques for the management of small-intermediate size JA. Resorting to external anterior or lateral approaches is still recommended in JAs encasing the ICA or with a massive feeder contribution from it, or in the rare instances of intradural spread.

## Figures and Tables

**Table 1:** 

Andrews et al. [52]	

(I)	Limited to the nasopharynx and nasal cavity. Bone destruction negligible or limited to the sphenopalatine foramen
(II)	Invading the pterygopalatine fossa or the maxillary, ethmoid, or sphenoid sinus with bone destruction
(III)	(a) Invading the infratemporal fossa or orbital region without intracranial involvement
	(b) Invading the infratemporal fossa or orbit with intracranial extradural (parasellar) involvement
(IV)	(a) Intracranial intradural without infiltration of the cavernous sinus, pituitary fossa or optic chiasm
	(b) Intracranial intradural with infiltration of the cavernous sinus, pituitary fossa or optic chiasm

Radkowski et al. [53]	

(I)	(A) Limited to posterior nares and/or nasopharyngeal vault
	(B) Involving the posterior nares and/or nasopharyngeal vault with involvement of at least one paranasal sinus
(II)	(A) Minimal lateral extension into the pterygopalatine fossa
	(B) Full occupation of pterygopalatine fossa with or without superior erosion orbital bones
	(C) Extension into the infratemporal fossa or extension posterior to the pterygoid plates
(III)	(A) Erosion of skull base (middle cranial fossa/base of pterygoids)—minimal intracranial extension
	(B) Extensive intracranial extension with or without extension into the cavernous sinus

Önerci et al. [54]	

(I)	Nose, nasopharyngeal vault, ethmoidal-sphenoidal sinuses, or minimal extension to PMF
(II)	Maxillary sinus, full occupation of PMF, extension to the anterior cranial fossa, and limited extension to the infratemporal fossa (ITF)
(III)	Deep extension into the cancellous bone at the base of the pterygoid or the body and the greater wing of sphenoid, significant lateral extension to the ITF or to the pterygoid plates posteriorly or orbital region, cavernous sinus obliteration
(IV)	Intracranial extension between the pituitary gland and internal carotid artery, tumor localization lateral to ICA, middle fossa extension, and extensive intracranial extension

Snyderman et al. [56]	

(I)	No significant extension beyond the site of origin and remaining medial to the midpoint of the pterygopalatine space
(II)	Extension to the paranasal sinuses and lateral to the midpoint of the pterygopalatine space
(III)	Locally advanced with skull base erosion or extension to additional extracranial spaces, including orbit and infratemporal fossa, no residual vascularity following embolisation
(IV)	Skull base erosion, orbit, infratemporal fossa
	Residual vascularity
(V)	Intracranial extension, residual vascularity
	M: medial extension
	L: lateral extension

**Table 2:** 

	Mohammadi Ardehali et al. [76]	Nicolai et al. [77]
Number of patients	47	46
Mean age (years)	17.1 (7–37)	17 (10–35)
Previous treatment	16	5
	(IA) 5	(IA) 3
	(IB) 10	(IB) 1
Stage (Radkowski classification)	(IIA) 3	(IIA) 5
	(IIB) 3	(IIB) 10
	(IIC) 22	(IIC) 19
	(IIIA) 3	(IIIA) 7
	(IIIB) 1	(IIIB) 1
Preoperative embolization	5	40
Mean blood loss (mL)	1336.2 (300–8500)	580 (250–1300)
Mean hospitalization time (days)	3.1	5
Mean followup (months)	33.1	73
Persistence rate (%)	19.1	8.6
